# PET quantification of [^18^F]MPPF in the canine brain using blood input and reference tissue modelling

**DOI:** 10.1371/journal.pone.0218237

**Published:** 2019-06-11

**Authors:** Glenn Pauwelyn, Lise Vlerick, Robrecht Dockx, Jeroen Verhoeven, Andre Dobbeleir, Kathelijne Peremans, Ingeborg Goethals, Tim Bosmans, Christian Vanhove, Filip De Vos, Ingeborgh Polis

**Affiliations:** 1 Laboratory of Radiopharmacy, Ghent University, Ghent, Belgium; 2 Small Animal Departments, Faculty of Veterinary Medicine, Ghent University, Merelbeke, Belgium; 3 Department of Psychiatry and Medical Psychology, Ghent University, Ghent, Belgium; 4 Department of Nuclear Medicine, Ghent University Hospital, Ghent, Belgium; 5 Institute Biomedical Technology–Medisip–Infinity, Ghent University, Ghent, Belgium; Wayne State University, UNITED STATES

## Abstract

Numerous studies have shown that the serotonin_1A_ (5-HT_1A_) receptor is implicated in the pathophysiology and treatment of several psychiatric and neurological disorders. Furthermore, functional imaging studies in a variety of species have demonstrated that 4-(2´-Methoxyphenyl)-1-[2´-(N-2´´-pyridinyl)-p- [^18^F]fluorobenzamidoethylpiperazine ([^18^F]MPPF) is a valid and useful PET tracer to visualize the 5HT_1A_ receptor. However, to our knowledge, [^18^F]MPPF has never been demonstrated in the canine brain. The ability to image the 5HT_1A_ receptor with PET in dogs could improve diagnosis and therapy in both canine and human behavioural and neuropsychiatric disorders. To examine the potential use of [^18^F]MPPF in dogs, five healthy adult laboratory beagles underwent a 60-minutes dynamic PET scan with [^18^F]MPPF while arterial blood samples were taken. For each region of interest, total distribution volume (V_T_) and corresponding binding potential (BP_ND_) were calculated using the 1-tissue compartment model (1-TC), 2-Tissue compartment model (2-TC) and Logan plot. The preferred model was chosen based on the goodness-of-fit, calculated with the Akaike information criterium (AIC). Subsequently, the BP_ND_ values of the preferred compartment model were compared with the estimated BP_ND_ values using three reference tissue models (RTMs): the 2-step simplified reference tissue model (SRTM2), the 2-parameter multilinear reference tissue model (MRTM2) and the Logan reference tissue model. According to the lower AIC values of the 2-TC model compared to the 1-TC in all ROIs, the 2-TC model showed a better fit. Calculating BP_ND_ using reference tissue modelling demonstrated high correlation with the BP_ND_ obtained by metabolite corrected plasma input 2-TC. This first-in-dog study indicates the results of a bolus injection with [^18^F]MPPF in dogs are consistent with the observations presented in the literature for other animal species and humans. Furthermore, for future experiments, compartmental modelling using invasive blood sampling could be replaced by RTMs, using the cerebellum as reference region.

## Introduction

The serotonin_1A_ (5-HT_1A_) receptor is a G-protein-coupled receptor and is believed to be one of the most important 5-HT receptor subtypes [1–3]. Two different 5-HT_1A_ receptor populations can be found in the mammalian brain [4–8]. The first population is located in the raphe nucleus and has an auto-receptor function that inhibits serotonin release at the nerve terminals. The second population is highly abundant in cortico-limbic areas, were it acts as post-synaptic receptor [2,9].

The 5-HT_1A_ receptor is involved in the pathophysiology of a variety of psychiatric and neurological disorders including depression, anxiety, schizophrenia, dementia, eating disorders, hallucinogenic behaviour, epilepsy and motion sickness [6,10–14]. Functional imaging studies of this receptor in healthy volunteers and in people suffering from neuropsychiatric disorders could improve diagnosis and therapy of these diseases. Therefore, several radioligands have been studied to assess *in vivo* changes in the 5HT_1A_ receptors using positron emission tomography (PET). Several PET-studies have demonstrated that 4-(2´-Methoxyphenyl)-1-[2´-(N-2´´-pyridinyl)-p- [^18^F]fluorobenzamidoethylpiperazine ([^18^F]MPPF) is a valid and reliable PET-tracer for imaging the 5HT_1A_ receptor in the human brain [10,15]. *In vitro* binding assays with the tritiated compound showed that this 5-HT_1A_ antagonist has a high affinity (Kd: 0.34 nM) and good selectivity towards the 5-HT_1A_ receptors [16]. Other advantages of this radiotracer are a high initial brain uptake, the relatively simple radiochemical synthesis compared to other 5HT_1A_ PET-tracers (for example: [^11^C]WAY100635) and the long physical half-life of ^18^F, which permits distribution from production facilities to remote hospitals [1,10,17]. To date, [^18^F]MPPF has been used successfully in rodents [18–21], cats [7,22,23], non-human primates [24,25] and humans [10,15,26–28]. Unfortunately, to the authors’ knowledge, it has never been used in dogs. However, functional imaging of the 5-HT_1A_ receptor in dogs could offer several opportunities as dogs exhibit naturally occurring behavioral disorders that may be comparable to certain human psychiatric conditions [29–33]. Therefore, dogs are considered as a valuable animal model for several human psychiatric disorders. Moreover, as opposed to rodents, dogs have a substantial amount of frontal cortex [34], which makes them a more interesting and more practical animal model in human psychiatry. Radioprotective regulations are also less stringent in dogs than in humans, allowing longitudinal studies with repeated PET scans in a relative short time frame. In addition, functional imaging studies of the 5-HT_1A_ receptor in dogs could also improve our understanding of several canine behavioral disorders and may serve as a tool to improve diagnosis and treatment of these disorders.

The objective of this study was to quantify dynamic [^18^F]MPPF PET data, obtained in healthy beagle dogs, in pharmacological parameters utilizing compartmental modeling. Several standard kinetic models were evaluated using metabolite corrected arterial plasma input functions and time-activity curves of each region of interest. Subsequently, to avoid invasive arterial blood sampling in future experiments and to improve the clinical applicability of the analysis method, the validity of different reference tissue models was assessed.

## Materials and methods

### Experimental animals

This study was approved by the Ethical committee of Ghent university (EC approval 17/108). Five neutered female experimental beagles (age 4.7 ± 0.1 years; weight 11.2 ± 0.7 kg) were included in this study. The animals were classified as healthy based on general clinical examination. They were provided by the faculty of veterinary medicine (Ghent University) and were socially-housed in small groups (2 to 8 dogs) on an internal surface of 15 m^2^ with permanent access to an outside area of 15 m^2^, equipped with enrichment tools. Food was withheld for at least 12 hours before anaesthesia but water was provided *ad libitum*. Prior to transportation to the PET centre of Ghent university hospital, the dogs were premedicated with an intramuscular (i.m.) injection of dexmedetomidine (375 μg/m^2^ body surface area, Dexdomitor, Orion Corporation, Espoo, Finland). At the PET centre, a 22G over-the-needle venous catheter was placed in one of the cephalic veins to gain intravenous access and a Lactated Ringer’s solution (Vetivex 500 mL, Dechra Veterinary products, Heusden-Zolder, Belgium) was infused i.v. at a rate of 5 mL/kg/h. General anaesthesia was induced with propofol (Propovet, Abbott laboratories, Queenborough, UK) given i.v. to effect. During the scan, anaesthesia was maintained with a mixture of 1.2–1.4% isoflurane (Isoflo, Abbott laboratories, Queenborough, UK) vaporized in oxygen using a circle rebreathing system. After positioning the dog on the bed of the PET/CT scanner, a 22G arterial catheter was placed in one of the dorsalis pedis arteries to perform arterial blood sampling. During general anaesthesia, a continuous monitoring of body temperature and cardiorespiratory functions by pulse oximeter and capnography was performed. After completion of the study, the dogs were reintroduced in their housing facility and remained available for future research.

### Radiosynthesis

[^18^F]MPPF was synthesized on a Synthra RN^+^ module (Synthra GmbH, Hamburg, Germany). Labelling precursor solution was prepared by dissolving five milligram nitro-MPPF (ABX, Radeberg, Germany) in 750 μL anhydrous DMSO (Sigma Aldrich, Germany). This was added to a dried [^18^F]F^-^/Kryptofix 222/K^+^ complex, heated at 150°C for 20 minutes, and diluted with 2.25 ml 0.05 M NaOAc (pH 5) after cooling down. Subsequently, HPLC purification was performed using a semipreparative HPLC system (Column: RP Symmetry Prep C18 (7 μm, 7.8 mm X 300 mm, Waters, Milford, Massachusetts, US); Solvent: 0.05 M NaOAc buffer pH 5/MeOH/THF: 50/32/18 (% V/V); flow: 3.5 mL/min). The [^18^F]MPPF fraction, eluted after 9 minutes, was collected during one minute. To remove the HPLC solvent, solid phase extraction (SPE) was performed using a C18 Sep-Pak cartridge (Braun, Germany), preconditioned with 10 mL ACN and 10 mL water. [^18^F]MPPF was eluted from the Sep-Pak by adding one millilitre of ethanol (VWR chemicals, Belgium). Finally, this ethanol eluate was diluted with nine millilitre of physiological saline (Braun, Germany) to obtain a formulation suitable for i.v. administration.

### Data acquisition

All dogs were scanned with a Siemens Biograph mCT Flow 20 clinical PET/CT imaging system (Siemens, Knoxville, USA). Prior to the PET scan, a low dose CT survey (120 kV, 35 mA, pitch of 0.7, 20 slices of 3 mm) was conducted and used for attenuation correction and anatomical framework. Dynamic PET recordings were initiated on bolus injection of 370 MBq [^18^F]MPPF. Emission data were reconstructed in 28 successive frames of increasing duration (6 x 10 s, 8 x 30 s, 5 x 120 s, 9 x 300 s), each consisting of a 512 x 512 matrix with a voxel size of 0.797 x 0.797 x 2 mm, using the TrueX algorithm. During the 60-minutes dynamic PET scan, arterial whole blood samples (1–2 mL) were taken manually into heparinized syringes at different time points (15, 30 and 45 seconds, 1, 1.5, 2, 4, 6, 8, 10, 15, 20, 30, 40 and 60 minutes) and collected in K_3_EDTA tubes. After centrifugation (10 min, 4000 rpm, 4°C) of the blood samples, the plasma fraction was separated from the blood cells. Radioactivity in plasma (100 μl) was measured using a gamma counter (Cobra, Canberra, Australia). The plasma parent compound fraction (%) was determined at six time points (15 seconds, 1, 4, 10, 20 and 40 minutes). Plasma fraction was mixed with 1:2 acetonitrile (Sigma Aldrich, Saint Louis, US) and centrifuged for 10 min at 4000 rpm. The resulting supernatant was then filtered using an Acrodisc Syringe filter 0.2 μm (Hoegaarden, Belgium) and injected (2 mL) onto the semipreparative HPLC system (Column: Symmetry Prep C18 column (7 μm, 7.8 mm X 300 mm, Waters, Milford, Massachusetts, US); Solvent: 0.05 M NaOAc buffer pH 5/MeOH/THF: 50/32/18 (V/V); flow: 3.5 mL/min). To calculate the parent compound fraction (%), 30 HPLC eluent fractions (each 0.5 min) were collected during a 15 minutes run and measured using a gamma counter (Cobra, Packard, Canberra, Australia).

### Image analysis

Prior to the PET scan, a series of T1 weighted anatomical images (3D MPRAGE sequence, 176 sagittal slices, TR: 2250 ms, TE: 4.18 ms, TI: 900 ms, parallel acquisition method = GRAPPA acceleration factor: 2, matrix size: 256 x 256, FOV: 220 mm, flip angle: 8°, voxel size: 1 x 1 x 1 mm^3^) were acquired using a 3T Magnetom Trio Tim system MRI scanner (Siemens, Erlangen, Germany). Subsequently, these images were co-registered with the PET/CT images using PMOD software version 3.405 (PMOD Technologies, Ltd., Zurich, Switzerland). Based on information from a dog brain atlas [35], 16 regions of interest (ROI) were manually delineated on the MR image. These included: left frontal cortex, right frontal cortex, left temporal cortex, right temporal cortex, left occipital cortex, right occipital cortex, left parietal cortex, right parietal cortex, midbrain, anterior cingulate gyrus, posterior cingulate gyrus, left hippocampus, right hippocampus, subgenual cingulate gyrus, presubgenual cingulate gyrus and cerebellum. Subsequently, time-activity curves could be obtained for each ROI representing the radioactivity concentration into each time frame, corrected for decay.

For each of the dogs, a Watabe function was fitted to the six metabolite time points and added to the plasma input function to obtain a metabolite-corrected plasma input function [36].

### Kinetic modelling

All kinetic modelling was performed using the kinetic tool of the PMOD software version 3.405. The total volume of distribution (V_T_), defined as the ratio of the concentration of radiotracer in a certain ROI to the concentration in plasma at equilibrium, was calculated for the different ROIs using the one- and two-tissue compartment (1-TC and 2-TC) model [37] and the Logan plot [38].

In order to validate that a reference tissue model constitutes as a good alternative for the invasive blood sampling, the non-displaceable binding potential (BP_ND_), referring to the ratio at equilibrium of specifically bound radioligand to that of non-displaceable (ND) radioligand in tissue, was estimated for both compartment models and the Logan plot [39]. Therefore, a region devoid of the targeted receptors (i.e. a reference region) is required. Various *in vitro* and *in vivo* experiments have demonstrated the absence of 5HT_1A_ receptors in the cerebellum, and therefore, the cerebellum has been put forward as reference region [19,39–41].

The goodness-of-fit for each compartment model was evaluated using the Akaike Information Criterion (AIC) value, where the lower the AIC value is, the better the model fits to the data [42]. For both compartmental models, the brain activity was corrected for the contribution of blood activity assuming a cerebral blood volume in the regions of interest fixed at 0.05 mL/cm^3^.

Furthermore, three RTMs were included in this study: the 2-step simplified reference tissue model (SRTM2) [43], the 2 parameter multilinear reference tissue model (MRTM2) [44] and the Logan reference tissue model [45]. For each of these models the possibility to reproduce the BP_ND_, obtained with the compartment models and the Logan plot, was analysed. Based on previous studies in humans [46] and rats [47], the cerebellum was used as a reference region. Furthermore, the BP_ND_ was determined using a fixed k_2_’ value based on the mean k_2_’ of four high binding regions: hippocampus left, hippocampus right, presubgenual cingulate gyrus and subgenual cingulate gyrus. For the SRTM2 model and Logan reference model, regional coupling with the SRTM2 model was used to calculate the k_2_’ value. For the MRTM2 model, the MRTM model was used to calculated the k_2_’ value.

### Test-retest analysis

To asses test-retest variability of the model parameters, one dog received two additional 60-minutes dynamic PET scans with bolus injection of 370 MBq [^18^F]MPPF. BP_ND_ were calculated using the three different RTMs (SRTM2, MRTM2 and Logan reference model) as described above.

### Statistical analysis

Statistical analysis was computed using RStudio 1.1.456 using packages MASS (version 7.3–50) and Sommer (version 3.0). To compare the BP_ND_ from the 2-TC models with those from the RTM, a multivariate linear mixed model with heterogeneous (unstructured) variances was set up on the data, containing BP_ND_ as response variable and the delineated ROI’s as outcome variable. The model included the individual animal as a random factor and the different kinetic models as fixed factor. The degrees of freedom were calculated based on the Welsh-Satterthwaite equation and the type-I error α was set at 0.001 (two-tailed) after Bonferroni correction (correction for the comparison between 15 regions and 4 different models). Furthermore, a random intercept was included. Finally, Pearson correlation coefficients (R^2^) were calculated between all models using Microsoft Excel.

## Results

### Radiosynthesis

The synthesis procedure of [^18^]MPPF gave rise to end of synthesis (EOS) activities of 1420 ± 650 MBq with high radiochemical and chemical purities of > 99%. The specific radioactivity measured with analytical HPLC was at least 101.3 GBq/μmol at EOS.

### Blood input function and metabolites

**Fig 1:**
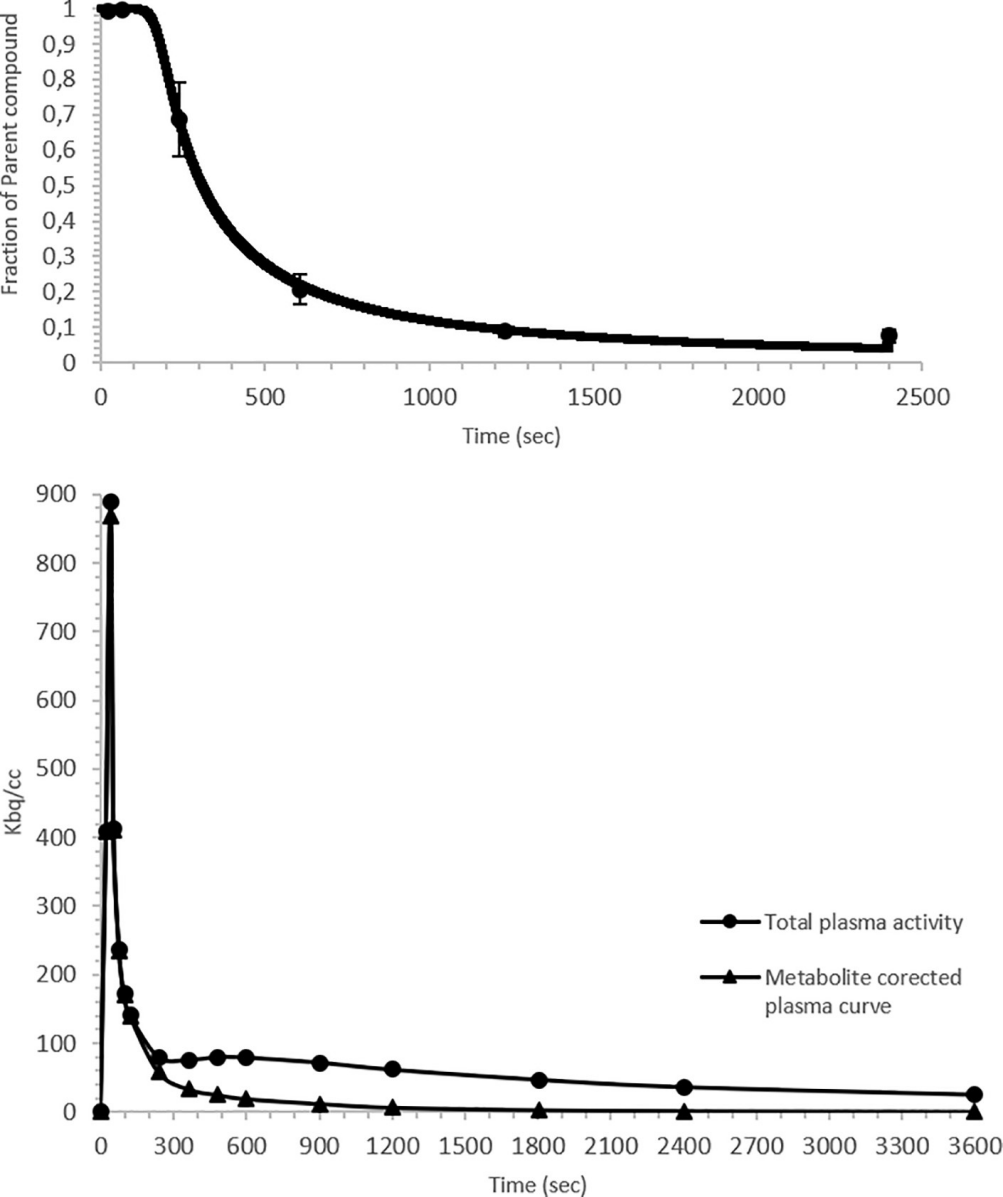
**A: Fraction (%) of parent compound in plasma over time.** The Watabe function fitted to the fraction of parent compound ([^18^F]MPPF) (mean ± SD) in plasma over time. **B: Metabolite corrected plasma input function**. A representative total plasma activity curve and corresponding metabolite corrected plasma curve.

Fig 1A represents the plasma parent compound fraction over time (averaged for five dogs). [^18^F]MPPF was rapidly metabolised to polar metabolites over the course of the PET scan. After 4 minutes, [^18^F]MPPF accounted for 69 ± 10% of total activity in the blood plasma, 21 ± 4% after 10 minutes and declined to 7 ± 2% at 40 minutes. A Watabe function could be fitted to the fraction of parent tracer in plasma for each individual dog. Subsequently, a metabolite-corrected plasma input curve could be calculated in PMOD (Fig 1B).

### Brain analysis

**Fig 2:**
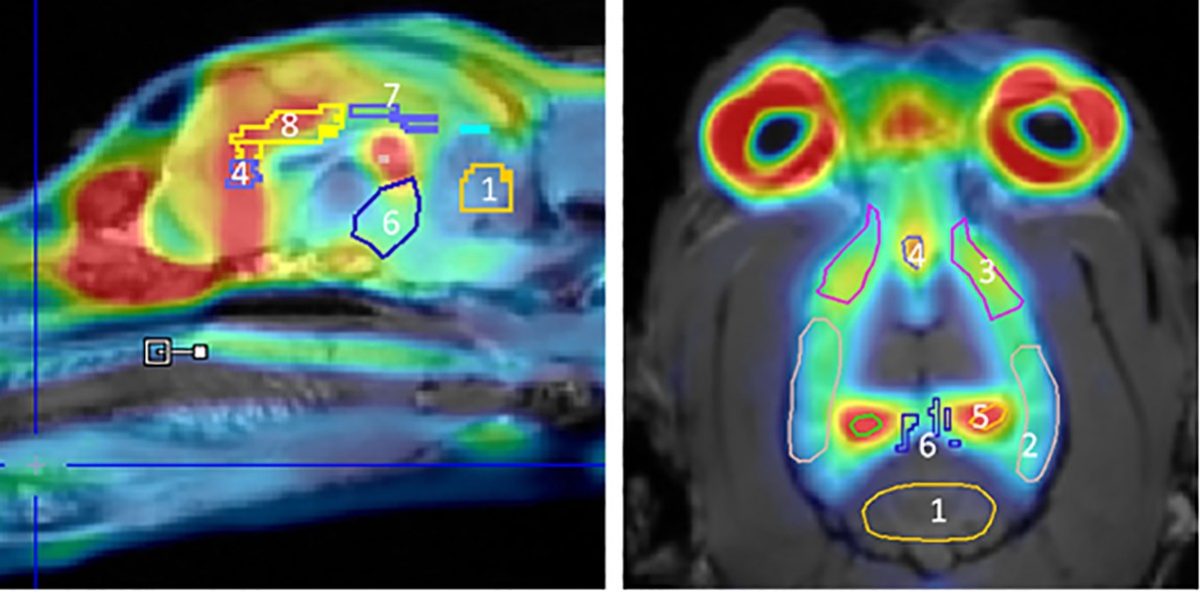
PET/MR image. Summed PET image (frames: 10 to 60 min) after bolus injection of [^18^F]MPPF co-registered with MR image. 1: cerebellum, 2: occipital cortex, 3: frontal cortex, 4: subgenual cingulate gyrus, 5: hippocampus, 6: midbrain, 7: posterior cingulate cortex (PCC) and 8: anterior cingulate cortex (ACC).

**Fig 3:**
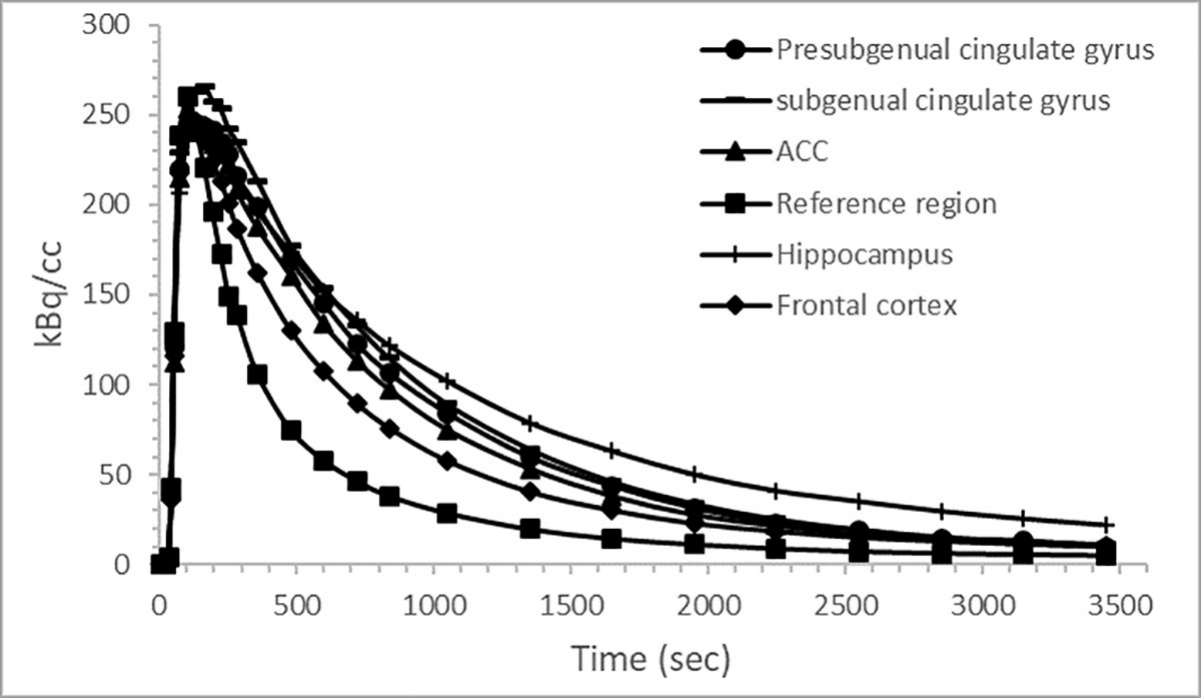
Time-activity curves. Regional time activity curves after bolus injection of [^18^F]MPPF for the presubgenual cingulate gyrus, subgenual cingulate gyrus, anterior cingulate cortex (ACC), hippocampus, frontal cortex and reference region. The data were corrected for radioactive decay.

Fig 2 represents a summed PET image co-registered with the MR image after bolus injection of [^18^F]MPPF. The highest radioactive uptake was found in the hippocampus, anterior cingulate cortex, presubgenual cingulate gyrus and subgenual cingulate gyrus. Intermediate radioactive uptake was found in the frontal cortex. The lowest radioactive uptake was observed in the cerebellum (i.e. the reference region). The corresponding time-activity curves are shown in Fig 3.

### Kinetic modelling

**Fig 4:**
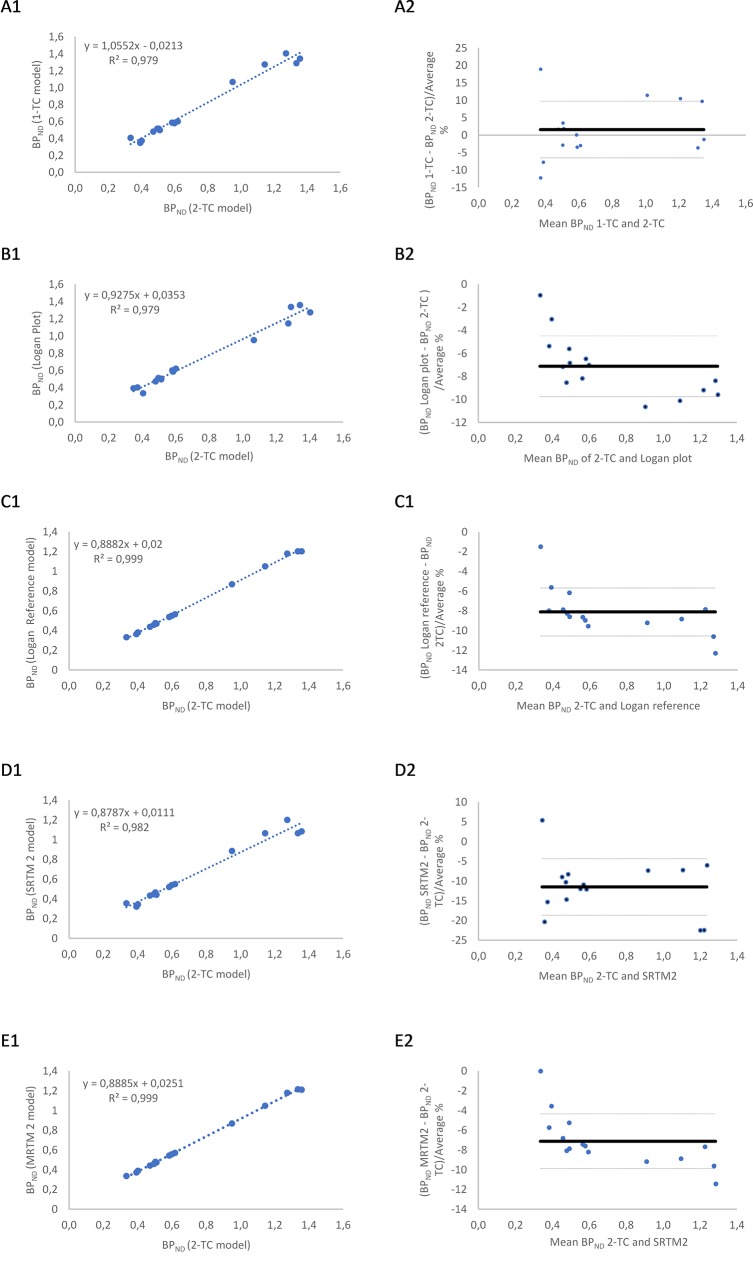
**(A-E). Method comparisons.** Graphical comparison of each kinetic model to the 2-TC model presented as a regression analysis (column 1) and a Bland and Altman plot were differences are presented as percentage (bold line: mean, dotted line: ± SD) (column 2). 4A: 1-TC model vs 2-TC model; 4B: 2-TC model vs Logan Plot; 4C: 2-TC model vs Logan reference model; 4D: 2-TC model vs SRTM2 model; 4E: 2-TC model vs MRTM2.

V_T_ values (mean + SD) and corresponding BP_ND_ in each ROI for the 1-TC and 2-TC model are shown in Table 1. The 1-TC and 2-TC model gave rise to maximum standard errors (SE) of 6.47% and 6.96%, respectively. The 2-TC model showed lower AIC-values in all of the observed brain regions compared to the 1-TC model. When plotting the BP_ND_ of the 1-TC model and 2-TC model (Fig 4A, column 2), no general over- or underestimation could be found, which is illustrated with a mean difference around zero in the Bland and Altman plot. In addition, the BP_ND_ obtained with the 1-TC and 2-TC model are well correlated with each other (R^2^ = 0.979) (Fig 4A, column 1). Furthermore, the V_T_ (SE_MAX_ = 1.29%) and corresponding BP_ND_ values obtained with the Logan plot are also shown in Table 1. BP_ND_-values obtained with the Logan plot were highly correlated with those from the 2-TC model (R^2^ = 0.979) (Fig 4B, column 1). Nevertheless, the Logan plot showed a moderate negative trend of differences, proportional to the magnitude of the measurement and mean underestimation of 7 ± 3% (Fig 4B, column 2).

**Table 1:** Kinetic parameters derived from 1-TC model, 2-TC model and Logan plot. Distribution volumes (V_T_), Akaike information criterion value (AIC) and binding potential (BP_ND_) in all the ROIs derived from plasma input data using the 1-TC, 2-TC model and Logan plot. The data is expressed as mean ± SD. BP_ND_ were estimated using the cerebellum as reference region, BP_ND_ = (V_T_/V_Ref_) -1.

	1-TC			2-TC			Logan Plot	
*Region*	V_T_	AIC	BP_ND_	V_T_	AIC	BP_ND_	V_T_	BP_ND_
*Presubgenual cingulate gyrus*	3.82 ± 1.03	52 ± 9	1.27 ± 0.29	4.35 ± 1.09	15 ± 23	1.15 ± 0.29	4.42 ± 1.06	1.03 ± 0.21
*Subgenual cingulate gyrus*	4.03 ± 1.05	51 ± 10	1.40 ± 0.30	4.61 ± 1.15	10 ± 23	1.27 ± 0.28	4.70 ± 1.10	1.16 ± 0.21
*Frontal cortex L*	2.67 ± 0.69	58 ± 10	0.59 ± 0.13	3.22 ± 0.76	21 ± 21	0.58 ± 0.15	3.34 ± 0.71	0.54 ± 0.12
*Frontal cortex R*	2.49 ± 0.58	58 ± 12	0.50 ± 0.19	3.04 ± 0.61	22 ± 21	0.51 ± 0.19	3.18 ± 0.57	0.48 ± 0.15
*Temporal cortex L*	2.56 ± 0.74	57 ± 10	0.51 ± 0.15	3.07 ± 0.82	21 ± 23	0.50 ± 0.13	3.22 ± 0.77	0.48 ± 0.11
*Temporal cortex R*	2.50 ± 0.69	56 ± 13	0.48 ± 0.15	3.00 ± 0.76	23 ± 20	0.47 ± 0.13	3.13 ± 0.71	0.44 ± 0.11
*Occipital cortex L*	2.33 ± 0.70	61 ± 12	0.37 ± 0.14	2.88 ± 0.84	26 ± 22	0.40 ± 0.11	3.04 ± 0.78	0.39 ± 0.09
*Occipital cortex R*	2.29 ± 0.70	62 ± 11	0.35 ± 0.15	2.85 ± 0.81	27 ± 21	0.39 ± 0.13	2.99 ± 0.74	0.37 ± 0.12
*Parietal cortex L*	2.69 ± 0.83	58 ± 11	0.58 ± 0.14	3.29 ± 0.94	23 ± 21	0.60 ± 0.10	3.43 ± 0.91	0.56 ± 0.08
*Parietal cortex R*	2.71 ± 0.82	57 ± 11	0.60 ± 0.16	3.32 ± 0.93	22 ± 21	0.62 ± 0.11	3.45 ± 0.87	0.58 ± 0.09
*Midbrain*	2.37 ± 0.59	49 ± 13	0.41 ± 0.10	2.71 ± 0.62	23 ± 20	0.34 ± 0.11	2.88 ± 0.57	0.33 ± 0.08
*ACC*	3.48 ± 0.96	50 ± 10	1.07 ± 0.26	3.96 ± 1.03	10 ± 21	0.95 ± 0.23	4.04 ± 0.99	0.85 ± 0.18
*PCC*	2.57 ± 0.78	55 ± 11	0.51 ± 0.20	3.06 ± 0.88	18 ± 24	0.50 ± 0.20	3.18 ± 0.82	0.46 ± 0.17
*Hippocampus R*	3.90 ± 0.79	61 ± 7	1.34 ± 0.26	4.73 ± 0.60	14 ± 24	1.36 ± 0.27	4.81 ± 0.84	1.23 ± 0.19
*Hippocampus L*	3.80 ± 0.82	64 ± 9	1.29 ± 0.32	4.69 ± 0.88	19 ± 19	1.34 ± 0.32	4.80 ± 0.95	1.23 ± 0.28
*Cerebellum*	1.71 ± 0.50	63 ± 14	-	2.06 ± 0.98	38 ± 23	-	2.19 ± 0.54	-

The BP_ND_ of [^18^F]MPPF estimated using the reference tissue methods (SRTM2, MRTM2 and Logan reference model) are shown in Table 2 and were plotted against those obtained with the 2-TC model (Fig 4C–4E). The BP_ND_ values obtained with reference tissue models showed SE < 10%. No significant difference could be found between each of the different reference tissue models and the 2-TC model in any of the ROI’s (p-values > 0.001), although, the Bland and Altman plots showed a mean underestimation of BP_ND_ ranging from 7 ± 3%(MRTM2) to 11 ± 7% (SRTM2). However, all models are highly correlated with the 2-TC model (R^2^(SRTM2) = 0.982, R^2^(Logan Reference) = 0.999, R^2^(MRTM2) = 0.999) (Fig 4C–4E). Moreover, a small enlargement correlating to the BP_ND_ levels for the Logan reference model and MRTM2 model could be seen in the Bland and Altman plots.

**Table 2:** BP_ND_ derived from the SRTM2-, Logan reference- and MRTM2 model. Binding potentials (BP_ND_) in all the ROIs derived from RTM using the SRTM2-, Logan reference- and MRTM2 model. The data is expressed as mean ± SD.

	SRTM2	Logan reference	MRTM2
*Region*	BP_ND_	BP_ND_	BP_ND_
*Presubgenual cingulate gyrus*	1.06 ± 0.22	1.05 ± 0.22	1.05 ± 0.21
*Subgenual cingulate gyrus*	1.20 ± 0.22	1.18 ± 0.21	1.18 ± 0.21
*Frontal cortex L*	0.52 ± 0.11	0.54 ± 0.12	0.54 ± 0.12
*Frontal cortex R*	0.44 ± 0.17	0.47 ± 0.16	0.47 ± 0.16
*Temporal cortex L*	0.46 ± 0.12	0.47 ± 0.11	0.48 ± 0.11
*Temporal cortex R*	0.43 ± 03.12	0.44 ± 0.11	0.44 ± 0.11
*Occipital cortex L*	0.35 ± 0.12	0.38 ± 0.10	0.39 ± 0.10
*Occipital cortex R*	0.32 ± 0.13	0.36 ± 0.12	0.37 ± 0.12
*Parietal cortex L*	0.53 ± 0.12	0.55 ± 0.10	0.56 ± 0.09
*Parietal cortex R*	0.54 ± 0.13	0.56 ± 0.11	0.57 ± 0.10
*Midbrain*	0.35 ± 0.08	0.33 ± 0.08	0.34 ± 0.08
*ACC*	0.88 ± 0.18	0.87± 0.18	0.87 ± 0.18
*PCC*	0.45 ± 0.17	0.45 ± 0.17	0.46 ± 0.16
*Hippocampus R*	1.08 ± 0.15	1.20 ± 0.19	1.21 ± 0.19
*Hippocampus L*	1.07 ± 0.15	1.20 ± 0.28	1.21 ± 0.27

### Test-retest variability

Test-retest variability for the regional BP_ND_ using three different RTMs is represented in Table 3. The mean percentage difference in BP_ND_ between test and retest ranges from 7.15% (SRTM2) to 8.56% (Logan reference). The lowest difference is seen in the cortical regions and hippocampus, while highest difference is seen in the presubgenual cingulate gyrus, ACC and midbrain.

**Table 3:** Test-retest variability. Binding potentials (BP_ND_) and % difference in all the ROIs derived from RTM using the SRTM2-, Logan reference- and MRTM2 model for the test-retest experiment.

	BP_ND_ (Test)			BP_ND_ (Retest)			% difference		
*Region*	SRTM2	MRTM2	Logan reference	SRTM2	MRTM2	Logan reference	SRTM2	MRTM2	Logan reference
*Presubgenual cingulate gyrus*	1.29	1.28	1.28	1.16	1.13	1.13	10.1	11.4	11.5
*Subgenual cingulate gyrus*	1.50	1.50	1.50	1.42	1.40	1.39	5.13	6.76	6.35
*Frontal cortex L*	0.75	0.76	0.76	0.70	0.73	0.72	6.66	4.78	4.65
*Frontal cortex R*	0.69	0.72	0.71	0.64	0.64	0.64	6.27	10.5	10.8
*Temporal cortex L*	0.64	0.65	0.65	0.61	0.67	0.66	3.83	2.73	3.85
*Temporal cortex R*	0.63	0.68	0.68	0.59	0.59	0.59	6.75	13.9	13.7
*Occipital cortex L*	0.42	0.45	0.45	0.39	0.42	0.41	6.77	7.67	7.88
*Occipital cortex R*	0.43	0.46	0.46	0.41	0.44	0.45	4.31	3.41	4.28
*Parietal cortex L*	0.63	0.65	0.65	0.61	0.67	0.67	3.70	4.04	3.79
*Parietal cortex R*	0.63	0.63	0.63	0.61	0.58	0.59	3.15	6.99	8.00
*Midbrain*	0.36	0.38	0.37	0.30	0.32	0.31	17.1	17.0	16.5
*ACC*	1.07	1.06	1.06	0.84	0.83	0.82	21.3	22.0	22.0
*PCC*	0.52	0.59	0.58	0.49	0.60	0.59	5.19	1.92	2.72
*Hippocampus R*	1.41	1.52	1.54	1.49	1.62	1.62	5.90	5.81	6.53
*Hippocampus L*	1.34	1.49	1.49	1.33	1.40	1.41	1.02	5.34	5.91
						**MEAN**	**7.15**	**8.27**	**8.56**

## Discussion

To our knowledge, this is the first study that examines the use of [^18^F]MPPF as PET-tracer to image the 5HT_1A_ receptor in the canine brain.

After a bolus injection of [^18^F]MPPF, high radioactive uptake was found in all brain regions followed by a rapid washout (Fig 3). The pattern of [^18^F]MPPF uptake into the brain corresponded well to the known distribution of the 5HT_1A_ receptor observed in human, rodent and cat studies [19,41,48]. The highest uptake of [^18^F]MPPF was found in the hippocampus, anterior cingulate cortex, presubgenual cingulate gyrus and subgenual cingulate gyrus. As expected, low uptake was found in the cerebellum, indicating an absence of 5HT_1A_ receptors as previous reported and further approving its use as reference region in RTMs (Fig 2) [19,40]. Because the location of the raphe nuclei in dogs is not clearly defined in the literature and was very difficult discernible on the PET image, the nuclei were not included in this study. In accordance to the clinical study of Costes et al. [28], a rapid metabolism of [^18^F]MPPF to polar metabolites was observed (Fig 1); 21 ± 4% of unmodified [^18^F]MPPF was found after ten minutes and declined to 7 ± 2% at 40 minutes. Due to limited sensitivity of the analysing method, the metabolite correction curve needed to be extrapolated from 40 to 60 minutes using the fitted Watabe function.

In this study, the 1-TC, 2-TC and Logan plot blood input models were compared with three RTMs: SRTM2, MRTM2 and Logan reference tissue model. According to the lower AIC values of the 2-TC model compared to the AIC values of 1-TC in all ROIs, the 2-TC model showed a better fit to the data, especially in the regions with high radioactive uptake: presubgenual cingulate gyrus and subgenual cingulate gyrus, hippocampus and cingulate cortex. The Logan plot showed very similar V_T_ values compared to the 2-TC model and the BP_ND_ were highly correlated with each other (R^2^ = 0.979), which indicates this graphical analysis could serve as a good alternative. Nevertheless, a moderate negative trend of differences and mean underestimation of 7 ± 3% should be kept in mind when using the Logan plot (Fig 4B, column 2).

Calculating BP_ND_, using RTM, with the cerebellum as reference region, showed high correlation with the BP_ND_ obtained by metabolite corrected plasma input 2-TC modelling (Fig 4). Although the Bland and Altman plots showed an underestimation of the BP_ND_ values when using the RTMs compared to the 2-TC model, no significant difference was found in any of the ROI’s. Furthermore, the MRTM2 model and reference Logan model show a dependency on the actual values. Nevertheless, the high correlation between the RTMs and 2-TC indicate the invasive blood sampling could be replaced by RTMs in future experiments with [^18^F]MPPF in dogs, which requires no arterial catheterisation or blood sampling. Based on the highest correlation values (R^2^ = 0.999) with the 2-TC model, the Logan reference model and MRTM2 model would be the models of choice, but a small enlargement correlating to the BP_ND_ levels should be mentioned for both models. An excellent alternative would be the SRTM2 model, which doesn’t have this dependency and showed also a very good correlation (R^2^ = 0.982) with the 2-TC model.

The test-retest variability was best for the SRTM2 model with a mean difference of 7.15%, compared to 8.27% and 8.56% for Logan reference model and the MRTM2 model, respectively. The cortical regions showed the lowest variability with a mean difference of 5.18%. High variability was seen in the presubgenuale gyrus and ACC for all three models. These regions are both very small which are highly susceptible to small errors in PET/MR fusion. High variability was also seen in the midbrain region but was due to very low BP_ND_-values.

Potential minor effects of the anaesthesia on the kinetics of [^18^F]MPPF during the PET-scan should be mentioned, although the use of anaesthesia is inevitable in dogs and a widely used anaesthesia protocol was used. Currently very little is known about the potential effects of various anaesthesia protocols on the kinetic of PET-tracers in dogs and should be further investigated in the future. Also the use of only female dogs in this study should be addressed. A second limitation of this study would be the absence of an *in vivo* blocking study, demonstrating the selectivity and specific binding of [^18^F]MPPF to the 5HT_1A_ receptor. However, this PET tracer has already proven its selectivity in various species and the high uptake brain regions are in accordance with other studies [19,41,48]. Furthermore, there is a 92% homology between the human 5HT_1A_ receptor and the canine 5HT_1A_ receptor [49]. Nevertheless, a future blocking study could provide a clear relationship between the model-derived parameters and the receptor expression levels.

## Conclusion

This study describes the first step in the visualisation and quantification of the 5HT_1A_ receptor using a bolus injection of [^18^F]MPPF in dogs. The results are consistent with the observations presented in the literature for other animal species and humans. The kinetics of [^18^F]MPPF in the canine brain could be best described by a 2-TC model. Furthermore, for future experiments, compartmental modelling using invasive blood sampling could be replaced by RTMs, using the cerebellum as reference region. This could be of great value for future experiments analysing the function of the 5HT_1A_ receptor, improving both diagnosis and therapy in canine and human behavioural and neuropsychiatric disorders.

## References

[1] Borg J. Molecular imaging of the 5-HT1A receptor in relation to human cognition. Behav Brain Res 2008;195:103–11. 10.1016/j.bbr.2008.06.011 18606193

[2] Hoyer D, Hannon JP, Martin GR. Molecular, pharmacological and functional diversity of 5-HT receptors. Pharmacol Biochem Behav 2002;71:533–54. 10.1016/S0091-3057(01)00746-8 11888546

[3] Aznavour N, Zimmer L. [18F]MPPF as a tool for the in vivo imaging of 5-HT1Areceptors in animal and human brain. Neuropharmacology 2007;52:695–707. 10.1016/j.neuropharm.2006.09.023 17101155

[4] Celada P, Puig MV, Amargós-bosch M, Adell A, Artigas F. CRSN Symposium: Focus on Depression, Part II Symposium du CRSN: le point sur la dépression, deuxième partie receptors in depression. J Psychiatry Neurosci JPN 2004;29:252–65. 15309042 PMC446220

[5] Garcia-Garcia AL, Newman-Tancredi A, Leonardo ED. P5-HT1Areceptors in mood and anxiety: Recent insights into autoreceptor versus heteroreceptor function. Psychopharmacology (Berl) 2014;231:623–36. 10.1007/s00213-013-3389-x 24337875 PMC3927969

[6] Savitz J, Lucki I, Drevets WC. 5-HT1A receptor function in major depressive disorder. Prog Neurobiol 2009;88:17–31. 10.1016/j.pneurobio.2009.01.009 19428959 PMC2736801

[7] Aznavour N, Rbah L, Riad M, Reilhac A, Costes N, Descarries L, et al A PET imaging study of 5-HT 1A receptors in cat brain after acute and chronic fluoxetine treatment 2006;33:834–42. 10.1016/j.neuroimage.2006.08.012 16996750

[8] Sharp T, Boothman L, Raley J, Quérée P. Important messages in the “post”: recent discoveries in 5-HT neurone feedback control. Trends Pharmacol Sci 2007;28:629–36. 10.1016/j.tips.2007.10.009 17996955

[9] Hannon J, Hoyer D. Molecular biology of 5-HT receptors. Behav Brain Res 2008;195:198–213. 10.1016/j.bbr.2008.03.020 18571247

[10] Passchier J, Van Waarde A. Visualisation of serotonin-1A (5-HT1A) receptors in the central nervous system. Eur J Nucl Med 2001;28:113–29. 10.1007/s002590000394 11202445

[11] Drevets WC, Frank E, Price JC, Kupfer DJ, Holt D, Greer PJ, et al PET imaging of serotonin 1A receptor binding in depression. Biol Psychiatr 1999;46:1375–87. 10.1016/s0006-3223(99)00189-4 10578452

[12] Elhwuegi AS. Central monoamines and their role in major depression. Prog Neuro-Psychopharmacology Biol Psychiatry 2004;28:435–51. 10.1016/j.pnpbp.2003.11.018 15093950

[13] Jans LAW, Riedel WJ, Markus CR, Blokland A. Serotonergic vulnerability and depression: Assumptions, experimental evidence and implications. Mol Psychiatry 2007;12:522–43. 10.1038/sj.mp.4001920 17160067

[14] Schreiber R. 5-HT1A receptor Ligands in Animal Models of Anxiety, Impulsivity and Depression: Multiple Mechanisms of Action? Prog Neuropsychopharmacol Biol Psychiatry 1993;17:87–104. 8416603 10.1016/0278-5846(93)90034-p

[15] Passchier J, van Waarde A, Pieterman RM, Elsinga PH, Pruim J, Hendrikse HN, et al Quantitative imaging of 5-HT(1A) receptor binding in healthy volunteers with [(18)f]p-MPPF. Nucl Med Biol 2000;27:473–6. 10.1016/s0969-8051(00)00114-1 10962253

[16] Kung HE, Stevenson DA, Zhuang Z, Kung M, Frederick D, Hurt SD. New 5-HT1A Receptor Antagonist: [3H]p-MPPF. Synapse 1996;23:344–6. 10.1002/(SICI)1098-2396(199608)23:4<344::AID-SYN13>3.0.CO;2-X 8855520

[17] Shiue C-Y, Shiue GG, Mozley PD, Kung M-P, Zhuang Z-P, Kim H-J, et al p- [18 F]-MPPF: A Potential Radioligand for PET Studies of 5-HT 1A Receptors in Humans. Synapse 1997;25:147–54. 10.1002/(SICI)1098-2396(199702)25:2<147::AID-SYN5>3.0.CO;2-C 9021895

[18] Lang L, Jagoda E, Schmall B, Vuong BK, Adams HR, Nelson DL, et al Development of fluorine-18-labeled 5-HT(1A) antagonists. J Med Chem 1999;42:1576–86. 10.1021/jm980456f 10229627

[19] Plenevaux A, Weissmann D, Aerts J, Lemaire C, Brihaye C, Degueldre C, et al Tissue distribution, autoradiography, and metabolism of 4-(2’-methoxyphenyl)-1-[2’ -[N-2"-pyridinyl)-p-[(18)F]fluorobenzamido]ethyl]piperazine (p-[(18)F]MPPF), a new serotonin 5-HT(1A) antagonist for positron emission tomography: An In vivo study in rats. J Neurochem 2000;75:803–11. 10.1046/j.1471-4159.2000.0750803.x 10899958

[20] Passchier J, Van Waarde A, Doze P, Elsinga PH, Vaalburg W. Influence of P-glycoprotein on brain uptake of F MPPF in rats. Eur J Pharmacol 2000;407:273–80. 10.1016/S0014-2999(00)00752-4 11068023

[21] Zimmer L, Rbah L, Giacomelli F, Bars D Le, Renaud B. A Reduced Extracellular Serotonin Level Increases the 5-HT1A PET Ligand 18F-MPPF Binding in the Rat Hippocampus. J Nucl Med 2003;44:1495–501. 12960198

[22] Aznavour N, Rbah L, Léger L, Buda C, Sastre JP, Imhof A, et al A comparison of in vivo and in vitro neuroimaging of 5-HT1Areceptor binding sites in the cat brain. J Chem Neuroanat 2006;31:226–32. 10.1016/j.jchemneu.2006.01.006 16517120

[23] Le Bars D, Lemaire C, Ginovart N, Plenevaux A, Aerts J, Brihaye C, et al High-yield radiosynthesis and preliminary in vivo evaluation of p- [18F]MPPF, a fluoro analog of WAY-100635. Nucl Med Biol 1998;25:343–50. 10.1016/S0969-8051(97)00229-1 9639295

[24] Shiue C-Y, Shiue GG, Mozley PD, Kung M-P, Zhuang Z-P, Kim H-J, et al p- [18 F]-MPPF: A Potential Radioligand for PET Studies of 5-HT 1A Receptors in Humans. Synapse 1997;25:147–54. 10.1002/(SICI)1098-2396(199702)25:2<147::AID-SYN5>3.0.CO;2-C 9021895

[25] Shively CA, Friedman DP, Gage HD, Bounds MC, Brown-Proctor C, Blair JB, et al Behavioral depression and positron emission tomography-determined serotonin 1A receptor binding potential in cynomolgus monkeys. Arch Gen Psychiatry 2006;63 10.1001/archpsyc.63.1.63 16585468

[26] Passchier J, Van Waarde A, Vaalburg W, Willemsen ATM. On the Quantification of [18 F]MPPF Binding to 5-HT 1A Receptors in the Human Brain. J Nucl Med 2001;42:1025–31. 11438622

[27] Costes N, Merlet I, Ostrowsky K, Faillenot I, Lavenne F, Zimmer L, et al A 18F-MPPF PET normative database of 5-HT1A receptor binding in men and women over aging. J Nucl Med 2005;46:1980–9. 46/12/1980 [pii]. 16330560

[28] Costes N, Merlet I, Zimmer L, Lavenne F, Cinotti L, Delforge J, et al Modeling [18F]MPPF positron emission tomography kinetics for the determination of 5-hydroxytryptamine(1A) receptor concentration with multiinjection. J Cereb Blood Flow Metab 2002;22:753–65. 10.1097/00004647-200206000-00014 12045674

[29] Overall KL. Natural animal models of human psychiatric conditions: assessment of mechanisms and validity. Prog Neuropsychopharmacol Biol Psychiatry 2000;24:727–76. 10.1016/S0278-5846(00)00104-4 11191711

[30] Cyranoski D. Pet Project. Nature 2010;466:1036–8. 10.1038/4661036a 20739982

[31] Vermeire S, Audenaert K, Dobbeleir A, de Meester R, Vandermeulen E, Waelbers T, et al Regional cerebral blood flow changes in dogs with anxiety disorders, measured with SPECT. Brain Imaging Behav 2009;3:342–9. 10.1007/s11682-009-9076-1

[32] Peremans K, Audenaert K, Coopman F, Blanckaert P, Jacobs F, Otte A, et al Estimates of regional cerebral blood flow and 5-HT2A receptor density in impulsive, aggressive dogs with99mTc-ECD and123I-5-I-R91150. Eur J Nucl Med Mol Imaging 2003;30:1538–46. 10.1007/s00259-003-1250-x 14579095

[33] Vermeire S, Audenaert K, De Meester R, Vandermeulen E, Waelbers T, De Spiegeleer B, et al Serotonin 2A receptor, serotonin transporter and dopamine transporter alterations in dogs with compulsive behaviour as a promising model for human obsessive-compulsive disorder. Psychiatry Res—Neuroimaging 2012;201:78–87. 10.1016/j.pscychresns.2011.06.006 22285716

[34] DeFelipe J. The Evolution of the Brain, the Human Nature of Cortical Circuits, and Intellectual Creativity. Front Neuroanat 2011;5:1–17. 10.3389/fnana.2011.00001 21647212 PMC3098448

[35] DuSharma S; Jacobs HL; Sharma K. The canine brain in stereotaxic coordinates: full sections in frontal, sagittal and hirzontal planes The MIT Press; 1970.

[36] Watabe H, Channing MA, Der MG, Adams R, Jagoda E, Herscovitch P, et al ll Kinetic Analysis of the 5-HT2A Ligand [C ] MDL 100, 907 2000:899–909. 10.1097/00004647-200006000-00002 10894173

[37] Schmidt KC, Turkheimer FE. Kinetic modeling in positron emission tomography. Q J Nucl Med 2002;46:70–85. 10.1016/B978-012744482-6.50026-0 12072847

[38] Logan J, Fowler JS, Volkow ND, Wolf AP, Dewey SL, Schlyer DJ, et al Graphical Analysis of Reversible Radioligand Binding from Time-Activity Measurements Applied to [N-11C-methyl]-(—)-Cocaine PET Studies in Human Subjects. Blood 1990:740–7. 10.1038/jcbfm.1990.127 2384545

[39] Innis RB, Cunningham VJ, Delforge J, Fujita M, Gjedde A, Gunn RN, et al Consensus nomenclature for in vivo imaging of reversibly binding radioligands. J Cereb Blood Flow Metab 2007;27:1533–9. 10.1038/sj.jcbfm.9600493 17519979

[40] Bars D Le, Lemaire C, Ginovart N, Plenevaux A, Aerts J, Brihaye C. High-Yield Radiosynthesis and Preliminary In Vivo Evaluation of p— [18 F ] MPPF, a Fluoro Analog of WAY-100635 1998;25:343–50. 10.1016/s0969-8051(97)00229-1 9639295

[41] Ginovart N, Hassoun W, Le Bars D, Weissmann D, Leviel V. In Vivo Characterization of p- [18 F]MPPF, a Fluoro Analog of WAY-100635 for Visualization of 5-HT 1A Receptors 2000;200:192–200. 10.1002/(SICI)1098-2396(20000301)35:3<192::AID-SYN4>3.0.CO;2-P 10657026

[42] Akaike H. A New Look at the Statistical Model Identification. IEEE Trans Automat Contr 1974;19:716–23. 10.1109/TAC.1974.1100705

[43] Wu Y, Carson RE. Noise reduction in the simplified reference tissue model for neuroreceptor functional imaging. J Cereb Blood Flow Metab 2002;22:1440–52. 10.1097/01.WCB.0000033967.83623.34 12468889

[44] Ichise M, Liow JS, Lu JQ, Takano A, Model K, Toyama H, et al Linearized reference tissue parametric imaging methods: Application to [11C]DASB positron emission tomography studies of the serotonin transporter in human brain. J Cereb Blood Flow Metab 2003;23:1096–112. 10.1097/01.WCB.0000085441.37552.CA 12973026

[45] Ziegler LD, Fan R, Desrosiers AE, Scherer NF. Distribution Volume Ratios Without Blood Sampling from Graphical Analysis of PET Data 1994;3:1823–39.

[46] Costes N, Zimmer L, Reilhac A, Lavenne F, Ryvlin P, Bars D Le, et al Test–Retest Reproducibility of 18 F-MPPF PET in Healthy Humans: A Reliability Study 2017:1279–89. 10.2967/jnumed.107.041905 17631552

[47] Millet P, Moulin M, Bartoli A, Del Guerra A, Ginovart N, Lemoucheux L, et al In vivo quantification of 5-HT1A-[18F]MPPF interactions in rats using the YAP-(S)PET scanner and a beta-microprobe. Neuroimage 2008;41:823–34. 10.1016/j.neuroimage.2008.02.062 18436452

[48] Sanabria-Bohórquez SM, Biver F, Damhaut P, Wikler D, Veraart C, Goldman S. Quantification of 5-HT1A receptors in human brain using p-MPPF kinetic modelling and PET. Eur J Nucl Med Mol Imaging 2002;29:76–81. 10.1007/s00259-001-0684-2 11807610

[49] Van Den Berg L, Versteeg SA, Van Oost BA. Isolation and Characterization of the Canine Serotonin Receptor IA Gene (htr1A). J Hered 2003;94:49–56. 10.1093/jhered/esg013 12692162

